# Supplementary material for: Molecular characterization of xerosis cutis: A systematic review
Source: PLoS One. 2021 Dec 16;16(12):e0261253. doi: 10.1371/journal.pone.0261253 (PMC8675746; doi:10.1371/journal.pone.0261253)
Supplement: S1 Appendix — (PDF) [file pone.0261253.s001.pdf]

Search Strategy; Molecular characterization of xerosis cutis:

Databases: 'MEDLINE','EMBASE' and 'Biological abstracts'.

Search conducted on: 29.09.2020

Xerosis cutis

1	(xerosis or xerotic).m_titl.
2	(astea* or xero*).m_titl.
3	(skin adj1 dry*).m_titl.
4	1 or 2 or 3
5	(subject or participa* or patient*).mp.
6	4 and 5

Markers

7	(biomarker or biologic* or new $marker or mediat* or express* or activat* or pathway or inflam*).mp.
8	(prote* or enzyme or cytokine or chemokine or IL$$ or TRL$ or TNF$ or inter*).mp.
9	(MMP$ or involucrin or Loricrin or prosta* or HAS$ or plako*).mp.
10	(corneodesmosin or aquaporin or filaggrin or keratin* or elasti*).mp.
11	(Lipid or ceramide or cholesterol or humec* or trigly* or endogen*).mp.
12	(Urocanic or Hyaluron* or malondialdehyde or glutathione or PCA$ or histamin or amino).mp.
13	7 or 8 or 9 or 10 or 11 or 12
14	6 and 13
15	Remove duplicates from 14
16	Limit 15 to yr= '1990 –Current'
