# Supplementary material for: Molecular characterization of xerosis cutis: A systematic review
Source: PLoS One. 2021 Dec 16;16(12):e0261253. doi: 10.1371/journal.pone.0261253 (PMC8675746; doi:10.1371/journal.pone.0261253)
Supplement: S2 Appendix — (DOCX) [file pone.0261253.s002.docx]

**S2 Appendix**

**Title: Study details and results of the data extraction of all included studies in the manuscript “Molecular characterization of xerosis cutis: a systematic review”**

**Authors: Ruhul Amin, Anna Lechner, Annika Vogt, Ulrike Blume-Peytavi, Jan Kottner.**

**Date: 20 October 2021.**

**Hanada et. al., 1984**

| Author | Hanada et. al. [23]  (Title: Relationship between dry skin and aluminium in hemodialysis patients.) |
| --- | --- |
| Year | 1984 |
| Study design | Cross sectional study |
| Country/ ethnicity | Japan (ethnicity: not reported) |
| Signs of dry skin and scoring method | Dry skin (hyperkeratosis, atrophy of eccrine sweat glands and stenosis of sweat ducts). |
| Analysed material | Epidermis and dermis |
| Sampling technique | Separation of the epidermis from the dermis (described in Baumberger et. al., 1942). |
| Method of analysis | Atomic absorption spectrophotometry. |

|  | Healthy volunteers (no further information about skin status) | Haemodialysis patients (with dry skin and sweat suppression) | |  | | Comments |
| --- | --- | --- | --- | --- | --- | --- |
| Number of participants | 8 | 5 | |  | |  |
| Age (mean) | Not reported | | |  | |  |
| Sex | Not reported | | |  | |  |
| Skin areas | Forearm | Forearm | |  | |  |
| Severity of dry skin | Normal skin (presumably) | Dry skin (strong suppression of sweating) | |  | |  |
| **Molecular marker** | | | Quan. units | |  | |
| Aluminium level in epidermis (mean, SD) | 55.6 (38.4) | 63.2 (20.3) | | µg/g tissue | |  |
|  | p value not reported | | |  |  |  |
| Aluminium level in dermis (mean, SD) | 5.6 (4.8) | 22.5 (12.3) | |  |  |  |
|  | p < 0.05 | | |  | |  |

**Saint-Léger et. al., 1988**

| Author | Saint-Léger et. al. [24]  (Title: Age-associated changes in stratum corneum lipids and their relation to dryness) |
| --- | --- |
| Year | 1988 |
| Study design | Cross sectional study |
| Country/ ethnicity | France and USA (ethnicity: Caucasian) |
| Signs of dry skin and scoring method | Dryness was visually evaluated according to a scale scoring from 0 to 4 as previously described in Kligman 1978.  Grade 0: Surface smooth (normal skin)  Grade 1: slight dryness; some sparse uplifted scales;  Grade 2: moderate dryness; uplifted scales more numerous.  Grade 3: extreme dryness; prominent large scales, densely covering the surface.  Grade 4: extreme dryness; prominent large scales, densely covering the surface with cracking and/or fissuring. |
| Analysed material | Compounds dissolved from stratum corneum. |
| Sampling technique | Using a turbine device containing a chamber placed on the skin in which 1 ml of hexane-methanol (2/3) was agitated for 1 min. |
| Method of analysis | Photodensitometry |

|  | Subjects with normal skin | Subjects with xerosis | | | | | |  | Comments |
| --- | --- | --- | --- | --- | --- | --- | --- | --- | --- |
| Number of participants | 50 in total | | | | | | |  |  |
| Age (mean) | Not reported | 25 | 35 | 45 | 55 | 65 | 75 |  |  |
| Sex | 45 females, 5 males | | | | | | |  |  |
| Skin areas | The lateral mid-calf | | | | | | |  |  |
| Severity of dry skin (grade) | Not reported | 1 | 1.2 | 2 | 2 | 1.5 | 3.1 |  | Quantity extracted from graph |
| **Molecular markers** | | | | | | | | Quan. units |  |
| Sterol esters (mean) | Not reported | 15.1 | 14.2 | 11.3 | 9.9 | 9.8 | 8.7 | Percentage | Quantity extracted from graph |
|  |  | r = -0.41 (p = 0.0037) | | | | | |  |  |
| Triglycerides (mean) | Not reported | 14.4 | 9.8 | 7.1 | 5.7 | 5.7 | 3.5 | Percentage | Quantity extracted from graph |
|  |  | r = -0.39 (p = 0.0002) | | | | | |  |  |
| Polar lipids (mean) | Not reported | 40.9 | 46.2 | 49.0 | 50.9 | 50.3 | 52.8 | Percentage | Quantity extracted from graph |
|  |  | r and p values not reported | | | | | |  |  |
| Increase in the index of sterol esterification (metric not reported) | Not reported | 0.48 | 0.54 | 0.60 | 0.62 | 0.64 | 0.66 | Index | Quantity extracted from graph |
|  |  | r and p values not reported | | | | | |  |  |
| Free Fatty Acid (metric not reported) | Not reported | 15 | 12.8 | 13.5 | 14.6 | 14.8 | 16.5 | Percentage | Quantity extracted from graph |
|  |  | r and p values not reported | | | | | |  |  |
| Cholesterol (metric not reported) | Not reported | 14.3 | 15.8 | 16.5 | 16.9 | 18.8 | 17.3 | Percentage | Quantity extracted from graph |
|  |  | r and p values not reported | | | | | |  |  |

**Horii et. al., 1989**

| Author | Horii et. al., [25]  (Title: Stratum corneum hydration and amino acid content in xerotic skin) |
| --- | --- |
| Year | 1989 |
| Study design | Part 1: cross sectional study  Part 2: pre-post study |
| Country/ ethnicity | Japan (ethnicity: not reported) |
| Signs of dry skin and scoring method | Senile xerosis was graded as follows:  Grade 0: normal appearing skin,  Grade 1: mild xerosis,  Grade 2: moderate xerosis,  Grade 3: severe xerosis. |
| Analysed material | Stratum corneum |
| Sampling technique | Serial adhesive tape-stripping (10 strippings). |
| Method of analysis | Amino acid analyser. |

**Study part 1**

|  | Subjects with normal skin | Subjects with mild xerosis | Subjects with moderate xerosis | Subjects with severe xerosis |  | Comments |
| --- | --- | --- | --- | --- | --- | --- |
| Number of participants | 7 | 10 | 8 | 5 |  |  |
| Age | 59 to 94 years | | | |  |  |
| Sex | Not mentioned | | | |  |  |
| Skin areas | The extensor surfaces of the lower leg | | | |  |  |
| Severity of dry skin | Grade 0 | Grade 1 | Grade 2 | Grade 3 |  |  |
| **Molecular marker** | | | | | Quan. units |  |
| Amino acid (metric not reported) | 1.22 | 0.96 | 0.89 | 0.55 | µmol/mg protein | Quantity extracted from graph |
|  | p < 0.05 | |  |  |  |  |
|  | p < 0.01 | | | |  |  |

**Study part 2**

|  | Pre-treatment | Post-treatment |  | Topical application of 10% urea-containing cream |
| --- | --- | --- | --- | --- |
| Number of participants | 10 | |  |  |
| Severity of dry skin | Average grade 2.3 | Average grade 0.2 |  |  |
| **Molecular marker** | | | Quan. units |  |
| Amino acid content (metric not reported) | 0.80 | 0.77 | µmol/mg protein | Quantity extracted from graph |
|  | p value not reported | |  |  |

**Saint-Léger et. al., 1989**

| Author | Saint-Léger et. al., [26]  (Title: Stratum corneum lipids in skin xerosis) |
| --- | --- |
| Year | 1989 |
| Study design | Cross sectional study |
| Country/ ethnicity | France and USA (ethnicity: not reported) |
| Signs of dry skin and scoring method | The dryness of skin was visually evaluated according to a scale scoring from 0 to 6 as previously described in Kligman 1978.  Grade 0: Normal; no sign of dryness  Grade 1: mild dryness; dusty, ashy appearance or an occasional minute skin flake.  Grade 2: mild dryness; dusty, ashy appearance or presence of many particles or minute skin flakes. Small lines or crevices were occasionally present filled with nondescript material.  Grade 3: moderate dryness with definite scaling (usually circular); borders of the scales were flat including characteristics of grade 1 and 2.  Grade 4: moderate dryness with well-defined scaling with raised edges; size of the scales were larger than in grade 3.  Grade 5: severe dryness with heavy scaling and/or fissuring. The scale plates were large with an increased lifting of the edges. Small fissures were occasionally seen between some scale plates. No erythema was evident.  Grade 6: Large scale plates with high lifting of the scale edges. Small fissures accompanied by erythema. |
| Analysed material | Compounds dissolved from stratum corneum |
| Sampling technique | Using a turbine device containing a chamber in which 5 ml of hexane-methanol (2/3) was agitated for 1 min. |
| Method of analysis | Photodensitometry |

|  | Subjects with normal skin | Subjects with xerosis (Grade 1 to 6) | | | | |  | Comments |
| --- | --- | --- | --- | --- | --- | --- | --- | --- |
| Severity of dry skin (grade) | 0 | 1 | 2 | 3 | 4 | 5 to 6 |  |  |
| Number of participants | 12 | None | 8 | 22 | 14 | 8 |  |  |
| Age | 30 to 40 years | | | | | |  |  |
| Sex | Females | | | | | |  |  |
| Skin areas | The outer aspect of the lower legs | | | | | |  |  |
| **Molecular markers** | | | | | | | Quan. units |  |
| Wax esters and sterol esters (mean, SE) | 17.8 (1.3) | Not reported | 14.4 (1.2) | 15 (1) | 14.3 (1) | 11.6 (1.2) | µg/cm^2^ |  |
|  |  |  | r = -0.31 (p = 0.027) | | | |  |  |
| Triglycerides (mean, SE) | 17 (1.8) | Not reported | 14.0 (2.3) | 14.7 (1.6) | 13.2 (1.7) | 9.6 (1.5) | µg/cm^2^ |  |
|  |  |  | r = -0.28 (p = 0.046) | | | |  |  |
| Free Fatty Acids (mean, SE) | 20.5 (2.8) | Not reported | 28.5 (3.2 | 27.7 (1.5) | 30.7 (1.5) | 41.5 (3.8) | µg/cm^2^ |  |
|  |  |  | r = +0.45 (p = 0.011) | | | |  |  |
| Free sterols (mean, SE) | 15.8 (1.0) | Not reported | 16.8 (1.85) | 15.9 (0.8) | 15.0 (1.0) | 14.0 (1.3) | µg/cm^2^ |  |
|  |  |  | r = -0.05 (p = 0.730) | | | |  |  |
| Ceramide I (mean, SE) | 1.74 (0.18) | Not reported | 2.2 (0.25) | 2.0 (0.3) | 1.75 (0.1) | 2.0 (0.26) | µg/cm^2^ |  |
|  |  |  | r =-0.09 (p = 0.540) | | | |  |  |
| Ceramide II (mean, SE) | 5.0 (0.54) | Not reported | 4.9 (0.4) | 4.75 (0.3) | 5.6 (0.7) | 4.7 (0.76) | µg/cm^2^ |  |
|  |  |  | r = +0.09 (p= 0.530) | | | |  |  |
| Ceramide III (mean, SE) | 6.8 (0.5) | Not reported | 6.8 (1.0) | 6.7 (0.5) | 5.8 (0.7) | 5.4 (0.6) | µg/cm^2^ |  |
|  |  |  | r = -0.09 (p = 0.550) | | | |  |  |
| Ceramide IV and V (mean, SE) | 6.25 (0.7) | Not reported | 5.75 (0.4) | 5.5 (0.3) | 5.6 (0.4) | 5.0 (0.3) | µg/cm^2^ |  |
|  |  |  | r = -0.06 (p = 0.660) | | | |  |  |
| Ceramide VI (mean, SE) | 6.6 (0.6) | Not reported | 5.4 (0.5) | 5.6 (0.35) | 6.0 (0.6) | 5.6 (0.4) | µg/cm^2^ |  |
|  |  |  | r = +0.06 (p = 0.660) | | | |  |  |
| Cholesteryl sulfate (mean, SE) | 2.8 (0.5) | Not reported | 1.6 (0.2) | 2.0 (0.17) | 2.0 (0.26) | 1.6 (0.2) | µg/cm^2^ |  |
|  |  |  | r = +0.07 (p = 0.670) | | | |  |  |
| Total Stratum corneum lipids (mean, SE) | 22.0 (1.8) | Not reported | 25.0 (2.5) | 22.8 (1.50) | 23.4 (1.9) | 26.3 (2.9) | µg/cm^2^ |  |
|  |  |  | r = -0.15 (p = 0.310) | | | |  |  |

**Jacobson et. al., 1990**

| Author | Jacobson et. al., [27]  (Title: Effects of aging and xerosis on the amino acid composition of human Skin.) |
| --- | --- |
| Year | 1990 |
| Study design | Cross sectional study |
| Country/ Ethnicity | USA (Ethnicity: Caucasian) |
| Signs of dry skin and scoring method | The dryness of the skin was not evaluated according to a clinical scale.  "Dry" refers to subjects diagnosed as having typical dry skin syndrome (xerosis), and "non-dry" refers to controls with skin judged to be normal. |
| Analysed material | Scraped cells from stratum corneum. |
| Sampling technique | An 8 x 8 cm area of the skin of each leg was scraped with a glass microscope slide and the cells were collected. |
| Method of analysis | High performance liquid chromatography. |

|  | Old subjects with non-dry skin | Old subjects with dry skin | Young subjects with dry skin | Young subjects with non-dry skin |  | Comments |
| --- | --- | --- | --- | --- | --- | --- |
| Number of participants | 7 | 13 | 8 | 18 |  |  |
| Age | 60 years or older | 60 years or older | 30 years or younger | 30 years or younger |  |  |
| Sex | Females | Females | Females | Females |  |  |
| Skin areas | The shins of both legs | The shins of both legs | The shins of both legs | The shins of both legs |  |  |
| Severity of dry skin | Normal | Dry skin (characterized by desquamating cells) | Dry skin (characterized by desquamating cells) | Normal |  |  |
| **Molecular markers** | | | | | Quan. units |  |
| Aspartic acid (mean) | 4.8 | 4.4 | 4.5 | 4.8 | Percent of total amino acids | Quantity extracted from graph |
|  | p value not reported | | | |  |  |
| Threonine (mean) | 6.4 | 5.9 | 6.4 | 6.2 |  |  |
|  | p ≤ 0.05 | |  |  |  |  |
|  |  | p ≤ 0.05 | |  |  |  |
| Serine (mean) | 28.7 | 28.2 | 27 | 26.7 |  |  |
|  | p value not reported | | | |  |  |
| Glutamic acid (mean) | 10.8 | 9.6 | 8.4 | 9.2 |  |  |
|  | p value not reported | | | |  |  |
| Glycine (mean) | 15.2 | 15.9 | 14.3 | 13.9 |  |  |
|  | p ≤ 0.05 | |  |  |  |  |
|  |  | p ≤ 0.05 | |  |  |  |
| Alanine (mean) | 9.1 | 8.9 | 7.6 | 8.2 |  |  |
|  |  | p ≤ 0.05 | |  |  |  |
| Valine (mean) | 3.0 | 3.0 | 2.9 | 2.9 |  |  |
|  | p value not reported | | |  |  |  |
| Methionine (mean) | 0.5 | 0.4 | 0.5 | 0.5 |  |  |
|  | p value not reported | | |  |  |  |
| Isoleucine (mean) | 1.6 | 1.7 | 1.9 | 1.8 |  |  |
|  | p value not reported | | |  |  |  |
| Leucine (mean) | 1.4 | 1.6 | 1.8 | 1.7 |  |  |
|  | p ≤ 0.05 | |  |  |  |  |
| Tyrosine (mean) | 1.6 | 1.8 | 2.0 | 1.7 |  |  |
|  | p ≤ 0.05 | |  |  |  |  |
| Phenylalanine (mean) | 0.9 | 1.0 | 1.0 | 1.0 |  |  |
|  | p ≤ 0.05 | |  |  |  |  |
| Lysine (mean) | 1.5 | 1.7 | 1.7 | 1.8 |  |  |
|  | p ≤ 0.05 | |  |  |  |  |
| Histidine (mean) | 6.6 | 6.2 | 6.5 | 7 |  |  |
|  | p value not reported | | | |  |  |
| Tryptophan (mean) | 2.4 | 2.8 | 3.0 | 3.3 |  |  |
|  | p value not reported | | | |  |  |
| Arginine (mean) | 1.5 | 1.5 | 2.2 | 2.1 |  |  |
|  | p value not reported | | | |  |  |
| Ornithine (mean) | 3 | 2.6 | 4.6 | 3.8 |  |  |
|  |  | p ≤ 0.05 | |  |  |  |

Note: Data of free amino acids from the water extract of skin sample. Data from soluble hydrolysate and whole cell hydrolysate were not extracted.

**Akimoto et. al., 1993**

| Author | Akimoto et. al. [28]  (Title: Quantitative analysis of stratum corneum lipids in xerosis and asteatotic eczema) |
| --- | --- |
| Year | 1993 |
| Study design | Cross sectional study |
| Country/ ethnicity | Japan (ethnicity: not reported) |
| Signs of dry skin and scoring method | Scoring method not reported.  In this study, "xerosis" was diagnosed as aged leg skin with dryness, itching and scales. The controls were healthy individuals who showed no dryness, scaling or itching in the winter season. |
| Analysed material | Stratum corneum. |
| Sampling technique | Stratum corneum sheet was removed from the skin area by a single stripping with cyanoacrylate resin. |
| Method of analysis | Thin layer chromatography. |

|  | Control (young) group | Age-matched control (older) group | Xerosis (older) group |  | Comments |
| --- | --- | --- | --- | --- | --- |
| Number of participants whose total lipids and total ceramides were analysed | 29 | 20 | 25 |  |  |
| Number of participants whose sebum-derived lipids (cholesterol ester, wax, triglyceride, free fatty acid, cholesterol sulfate) were analysed | 15 | 11 | 18 |  |  |
| Age of participants whose total lipids and total ceramides were analysed (metric not reported) | 24.3 years | 71.6 years | 71.0 years |  |  |
| Age of participants whose sebum-derived lipids (cholesterol ester, wax, triglyceride, free fatty acid, cholesterol sulfate) were analysed (metric not reported) | 27 years | 72 years | 71.4 years |  |  |
| Sex | Not reported | Not reported | Not reported |  |  |
| Skin areas | The extensor surfaces of the lower legs | The extensor surfaces of the lower legs | The extensor surfaces of the lower legs |  |  |
| Severity of dry skin | No dryness, scaling or itching | No dryness, scaling or itching | Skin with dryness, itching and scales |  |  |
| **Molecular markers** | | | | Quan. units |  |
| Total lipid (metric not reported) | 76.9 | 49.9 | 62.2 | µg/mg | -Quantity extracted from graph. |
|  | p < 0.01 | | |  |  |
| Total ceramide (metric not reported) | 18.3 | 12.2 | 15.7 |  |  |
|  |  | p < 0.01 |  |  |  |
| Ceramide 1 (metric not reported) | 0.91 | 0.63 | 0.86 |  |  |
|  | P value not reported | | |  |  |
| Ceramide 2 (metric not reported) | 3.20 | 2.11 | 3.31 |  |  |
|  |  | p < 0.01 | |  |  |
| Ceramide 3 (metric not reported) | 3.46 | 1.89 | 2.69 |  |  |
|  |  | p < 0.01 |  |  |  |
| Hydro- ceramide 1 (metric not reported) | 0.63 | 0.41 | 0.54 |  |  |
|  | p value not reported | | |  |  |
| Ceramide 4 and 5 (metric not reported) | 3.20 | 2.97 | 3.35 |  |  |
|  | p < 0.01 | | |  |  |
| Ceramide 6 (metric not reported) | 5.52 | 3.79 | 4.97 |  |  |
|  |  | p < 0.05 | |  |  |
| Cholesterol sulfate (metric not reported) | 3.94 | 2.48 | 3.26 |  |  |
|  | p < 0.05 | | |  |  |
| Cholesterol ester (metric not reported) | 1.0 | 0.9 | 0.8 |  |  |
|  | p value not reported | | |  |  |
| Wax (metric not reported) | 3.20 | 0.9 | 0.6 |  |  |
|  | p value not reported | | |  |  |
| Triglyceride (metric not reported) | 3.20 | 3.1 | 3.8 |  |  |
|  |  | p < 0.01 | |  |  |
| Free fatty acid (metric not reported) | 6.8 | 8.7 | 6.2 |  |  |
|  |  | p < 0.01 | |  |  |
| Cholesterol (metric not reported) | 3.20 | 2.8 | 3.1 |  |  |
|  | p value not reported | | |  |  |

**Park et. al., 1995**

| Author | Park et.al. [29]  (Title: Dry skin (xerosis) in patients undergoing maintenance haemodialysis: the role of decreased sweating of the eccrine sweat gland) |
| --- | --- |
| Year | 1995 |
| Study design | Case control |
| Country/ Ethnicity | Country: Korea (Ethnicity: Not reported) |
| Signs of dry skin and scoring method | 1. Normal skin.  2. Dry skin (appearing rough with or without scaling), 10 patients had pruritus (56%), while eight patients did not (44%). |
| Analysed material | Stratum corneum. |
| Sampling technique | Cyanoacrylate adhesive was attached to a defined area of 2.5 cm^2^on the ventral forearm and the horny layer was stripped off. |
| Method of analysis | Spectrophotometry. |

|  | Healthy volunteers (no further information about skin status) | Patients with xerotic skin undergoing maintenance haemodialysis |  | Comments |
| --- | --- | --- | --- | --- |
| Number of participants | 10 | 18 |  |  |
| Age  (mean) | 55 years  Age range: 41 to 62 years | 50 years  Age range: 30 to 68 years |  |  |
| Sex | 4 Males, 6 Females | 10 Males, 8 Females |  |  |
| Skin areas | Ventral forearm | Ventral forearm |  |  |
| Severity of dry skin | Normal skin (presumably) | Dry skin |  |  |
| **Molecular marker** | | | Quan. units |  |
| Urea (mean) | 5.04 | 28.2 | µg/cm^2^ |  |
|  | P < 0.05 | |  |  |

**Rawlings et. al., 1996**

| Author | Rawlings et al., [30]  Title: Effect of lactic acid isomers on keratinocyte ceramide synthesis, stratum corneum lipid levels and stratum corneum barrier function. |
| --- | --- |
| Year | 1996 |
| Study design | Double blind paired-comparison study (first study) |
| Country/ ethnicity | USA (ethnicity: Caucasian) |
| Signs of dry skin and scoring method | Clinical dryness and erythema scoring on a scale of 0 to 4.0. |
| Analysed material | Stratum corneum. |
| Sampling technique | Tape stripping (8 consecutive strips were collected with adhesive tapes) |
| Method of analysis | For lipids: densitometric analysis using a densitometer,  For fatty acid methyl esters (FAMEs): gas chromatography.  For protein: plate reading technique. |

|  | Control | Treatments | | |  | Comments |
| --- | --- | --- | --- | --- | --- | --- |
|  | Vehicle treated skin | D-Lactic acid treated skin | D, L-Lactic acid treated skin | L-Lactic acid treated skin |  | Treatment: 4% active formulations. |
| Number of participants | 24 subjects with dry skin | 6 Subjects (Same participants from control) | 6 Subjects (Same participants from control) | 12 Subjects (Same participants from control) |  | Treatment duration 4 weeks. |
| Age | 23 to 45 years | Same participants | Same participants | Same participants |  |  |
| Sex | Male and female | Same participants | Same participants | Same participants |  |  |
| Skin areas | Volar surface of one forearm | Volar surface of contralateral forearm | Volar surface of contralateral forearm | Volar surface of contralateral forearm |  |  |
| Severity of dry skin at baseline | No greater than 1.0 | | | |  |  |
| Severity of dry skin after 4 weeks | Not reported | | | |  |  |
|  | **After 4 weeks treatment** | | | |  |  |
| **Molecular markers** | | | | | Quan. Units |  |
| Increase in ceramide level (metric not reported) | Not reported | 0% | 25% | 38% | Percentage | Quantity extracted from graph |
|  | p value not reported | | | |  |  |
| Cholesterol levels (metric not reported) | 16.9 | Not reported | Not reported | 18.8 | ng lipid/μg protein | Quantity extracted from graph |
|  | p value not reported | | | |  |  |
| Fatty acid levels  (metric not reported) | 35.8 | Not reported | Not reported | 42.3 | ng lipid/μg protein | Quantity extracted from graph |
|  | p value not reported | | | |  |  |
| Ceramide levels  (metric not reported) | 27.6 | Not reported | Not reported | 38.8 | ng lipid/μg protein | Quantity extracted from graph |
|  | p < 0.05 | | | |  |  |
| Total ceramide 1 esterified fatty acid (16:0) ; metric not reported | 16.7 | Not reported | Not reported | 23.5 | Percentage | Quantity extracted from graph |
|  | p < 0.05 | | | |  |  |
| Total ceramide 1 esterified fatty acid (18:0) ; metric not reported | 26.4 | Not reported | Not reported | 21.2 | Percentage | Quantity extracted from graph |
|  | p value not reported | | | |  |  |
| Total ceramide 1 esterified fatty acid (18:1) ; metric not reported | 35.9 | Not reported | Not reported | 28.1 | Percentage | Quantity extracted from graph |
|  | p < 0.05 | | | |  |  |
| Total ceramide 1 esterified fatty acid (18:2) ; metric not reported | 17.9 | Not reported | Not reported | 24.3 | Percentage | Quantity extracted from graph |
|  | p value not reported | | | |  |  |
| Total ceramide 1 esterified fatty acid (20:0); metric not reported | 4.9 | Not reported | Not reported | 5.5 | Percentage | Quantity extracted from graph |
|  | p value not reported | | | |  |  |
| Improvement in ratio of ceramide 1 linoleate to ceramide 1 oleate (metric not reported) | 0.51 | Not reported | Not reported | 0.83 | Ratio | Quantity extracted from graph |
|  | p < 0.05 | | | |  |  |

**Schreiner et. al., 2000**

| Author | Schreiner et. al., [31]  (Title: Barrier characteristics of different human skin types investigated with x-ray diffraction, lipid analysis and electron microscopy imaging.) |
| --- | --- |
| Year | 2000 |
| Study design | Cross sectional study |
| Country/ ethnicity | Germany and the Netherlands (ethnicity: Caucasian) |
| Signs of dry skin and scoring method | The dryness of the skin was visually evaluated according to a clinical scale.  Visual score of scaliness:  1= no scale,  4= very scaly.  Sensory score of suppleness:  1= very smooth,  7= extremely rough. |
| Analysed material | Stratum corneum |
| Sampling technique | Shave biopsy |
| Method of analysis | High performance thin layer chromatography and photodensitometry. |

|  | Young with normal skin | Young with dry skin | Skin of aged participants |  | Comments |
| --- | --- | --- | --- | --- | --- |
| Number of participants | 10 | 5 | 4 |  |  |
| Age | 25.5 (SD 2.5) years | 30 (SD 6) years | 66 (SD 3) years |  |  |
| Sex | Not reported | Not reported | Not reported |  |  |
| Skin areas | The skin of the lower leg | The skin of the lower leg | The skin of the lower leg |  |  |
| Severity of dry skin (scaliness score)  (mean, SD) | 0.7 (0.5) | 3.1 (0.2 ) | 2.5 (1.2) |  |  |
| Severity of dry skin (suppleness score)  (mean, SD) | 2.5 (0.8) | 6.0 (0.4) | 4.5 (0.8) |  |  |
| **Molecular markers** | | | | Quan. units |  |
| Total ceramide  (mean, SEM) | 21 (4) | 20 (2) | 26 (11) | µg lipid per mg SC protein |  |
|  | p value not reported | | |  |  |
| Free sterols  (mean, SEM) | 17 (4) | 15 (1) | 23 (6) |  |  |
|  | p value not reported | | |  |  |
| Free fatty acids  (mean, SEM) | 17 (3) | 18 (5.5) | 38 (12) |  |  |
|  | p value not reported | | |  |  |
| Ceramide (EOS)/ total ceramide  (mean, SD) | 0.08 (0.03) | 0.09 (0.05) | 0.10 (0.05) | Quantity of analysed ceramide /quantity of total ceramide |  |
|  | p value not reported | | |  |  |
| Ceramide (NS)/ total ceramide  (mean, SD) | 0.16 (0.03) | 0.21 (0.01) | 0.17 (0.03) |  |  |
|  | p < 0.01 | |  |  |  |
| Ceramide (NP)/ total ceramide  (mean, SD) | 0.18 (0.04) | 0.16 (0.02) | 0.16 (0.04) |  |  |
|  | p value not reported | | |  |  |
| Ceramide (EOH)/ total ceramide (mean, SD) | 0.08 (0.02) | 0.07 (0.04) | 0.05 (0.03) |  |  |
|  | p value not reported | | |  |  |
| Ceramide (AS)/ total ceramide  (mean, SD) | 0.21 (0.02) | 0.23 (0.04) | 0.23 (0.02) |  |  |
|  | p value not reported | | |  |  |
| Ceramide (AP)/ total ceramide  (mean, SD) | 0.10 (0.02) | 0.07 (0.02) | 0.11 (0.01) |  |  |
|  | p value not reported | | |  |  |
| Ceramide (AH)/ total ceramide  (mean, SD) | 0.18 (0.03) | 0.18 (0.02) | 0.19 (0.01) |  |  |
|  | p value not reported | | |  |  |

**Simon et. al., 2001**

| Author | Simon et. al. [32]  (Title: Persistence of both peripheral and non-peripheral corneodesmosomes in the upper stratum corneum of winter xerosis skin versus only peripheral in normal skin) |
| --- | --- |
| Year | 2001 |
| Study design | Case control |
| Country/ ethnicity | Country: France (ethnicity: Caucasians) |
| Signs of dry skin and scoring method | 1. Normal skin.  2. Moderate to well-defined xerosis, i.e. dry skin characterized by roughness and papyraceous appearance of the skin, presence of raised squames and/or scales, and irritation. |
| Analysed material | Stratum corneum. |
| Sampling technique | Superficial stratum corneum extracts were obtained from the volunteers by three consecutive varnish-stripping (following Guerrin et al, 1998). |
| Method of analysis | Protein concentrations: protein assay (SDS-PAGE, western blotting). The immunoblotting reactivities, related to the detectable amounts of proteins, were quantified by densitometry using a software.  Corneodesmosome density (corneodesmosome area divided by total area): transmission electron microscopy. |

|  | Normal skin | Xerotic skin |  | Comments |
| --- | --- | --- | --- | --- |
| Number of participants | n=26 | n= 30 |  |  |
| Age | 22 to 49 years | 22 to 49 years |  |  |
| Sex | Females | Females |  |  |
| Skin areas | External parts of the legs | External parts of the legs |  |  |
| Severity of dry skin | Normal skin | Moderate to well-defined xerosis |  |  |
| **Molecular markers** | | | Quan. units |  |
| Desmoglein 1 (median) | 11.1 | 27.1 | Arbitrary scale | Quantity extracted from graph.  Amount was elevated in xerotic skin |
|  | p < 0.02 | |  |  |
| Plakoglobin (median) | 18.5 | 31.1 | Arbitrary scale | Quantity extracted from graph.  Amount was elevated in xerotic skin. |
|  | p < 0.02 | |  |  |
| Corneodesmosin (median) | 16.1 | 21.3 | Arbitrary scale | Quantity extracted from graph.  Amount was elevated in xerotic skin. |
|  | p = 0.05 | |  |  |
| Corneodesmosome density in the inner SC (n=2); metric not reported | 30.0 | 34.6 | Corneodesmosome surface/ µm^2^ (arbitrary scale) | Quantity extracted from graph. |
|  | p value not reported | |  |  |
| Corneodesmosome density in the outer SC (n= 3); metric not reported | 2.3 | 19.2 |  | Quantity extracted from graph.  Amount was elevated in xerotic skin. |
|  | p < 0.001 | |  |  |

**Takahashi et. al., 2004**

| Author | Takahashi et. al., [33]  (Title: The content of free amino acids in the stratum corneum is increased in senile xerosis.) |
| --- | --- |
| Year | 2004 |
| Study design | Cross sectional study |
| Country/ Ethnicity | Japan (Ethnicity: Not reported) |
| Signs of dry skin and scoring method | Not reported |
| Analysed material | Stratum corneum |
| Sampling technique | Several layers of the stratum corneum were scraped off with a knife or glass microscope slide and stratum corneum cells were collected. |
| Method of analysis | High performance liquid chromatography |

|  | Aged senile xerosis | Aged normal group | Young group |  | Comments |
| --- | --- | --- | --- | --- | --- |
| Number of participants | 12 | 5 | 10 |  |  |
| Age | 60 to 81 years | 60 to 74 years | 18 to 29 years |  |  |
| Sex | Not reported | Not reported | Not reported |  |  |
| Skin areas | The skin of the lower leg | The skin of the lower leg | The skin of the lower leg |  |  |
| Severity of dry skin | Not reported | Not reported | Not reported |  |  |
| **Molecular markers** | | | | Quan. units |  |
| Total Amino acid (metric not reported) | 581.4 | 497.7 | 322.4 | Pmol/ 1000 SC cells | -Quantity extracted from graph |
|  | p < 0.01 | | |  |  |
| Aspartic acid (metric not reported) | 31.5 | 29.0 | 10.8 |  |  |
|  | p value not reported | | |  |  |
| Glutamic acid (metric not reported) | 8.0 | 7.0 | 4.0 |  |  |
|  | p value not reported | | |  |  |
| Citrulline (metric not reported) | 54.5 | 48.0 | 43.9 |  |  |
|  | p value not reported | | |  |  |
| Serine (metric not reported) | 132.0 | 116.2 | 64 |  |  |
|  | p < 0.05 | | |  |  |
| Threonine (metric not reported) | 37.4 | 27.5 | 24 |  |  |
|  | p < 0.05 | | |  |  |
| Arginine (metric not reported) | 13.0 | 23.0 | 8.0 |  |  |
|  | p value not reported | | |  |  |
| Glycine (metric not reported) | 102.5 | 118.8 | 52.5 |  |  |
|  | p < 0.05 | | |  |  |
| Alanine (metric not reported) | 45.0 | 39.8 | 26.0 |  |  |
|  | p < 0.05 | | |  |  |
| Proline (metric not reported) | 14.3 | 11.0 | 8.0 |  |  |
|  | p value not reported | | |  |  |
| Valine (metric not reported) | 20.2 | 15.0 | 9.0 |  |  |
|  | p value not reported | | |  |  |
| Isoleucine (metric not reported) | 15.6 | 11.0 | 6.9 |  |  |
|  | p < 0.05 | | |  |  |
| Leucine (metric not reported) | 9.7 | 6.9 | 6.9 |  |  |
|  | p value not reported | | |  |  |
| Tryptophan (metric not reported) | 5.5 | 6.0 | 3.5 |  |  |
|  | p value not reported | | |  |  |
| Phenylalanine (metric not reported) | 6.0 | 5.2 | 2.9 |  |  |
|  | p < 0.05 | | |  |  |
| Urocanic acid (metric not reported) | 20.0 | 4.8 | 12.0 |  |  |
|  | p value not reported | | |  |  |
| Ornithine (metric not reported) | 14.0 | 4.8 | 4.8 |  |  |
|  | p < 0.05 | | |  |  |
| Lysine (metric not reported) | 9.7 | 7.2 | 5.3 |  |  |
|  | p < 0.05 | | |  |  |
| Histidine (metric not reported) | 33.2 | 36.0 | 17.6 |  |  |
|  | p < 0.05 | | |  |  |
| Tyrosine (metric not reported) | 12.2 | 11.4 | 6.0 |  |  |
|  | p < 0.05 | | |  |  |

**Delattre et. al., 2012**

| Author | Delattre et. al. [34] (Title: Proteomic analysis identifies new biomarkers for postmenopausal and dry skin) |
| --- | --- |
| Year | 2012 |
| Study design | Cross sectional |
| Country/ ethnicity | France and Canada (ethnicity: Caucasian) |
| Signs of dry skin and scoring method | 0: normal skin – regular cutaneous relief and smooth aspect;  1: dehydrated skin –streaked cutaneous relief and rather rough aspect;  2: dry skin – streaked cutaneous relief, some scales and rough aspect;  3: very dry skin – numerous scales and rough aspect;  4: extremely dry skin – very numerous scales and very rough aspect. |
| Sample | Stratum corneum |
| Sampling technique | Varnish stripping sampling |
| Method of analysis | (2D) Electrophoresis, western blot, liquid chromatography mass spectrometry. |

|  | Normal skin | Dry Skin |  | Comments |
| --- | --- | --- | --- | --- |
| Number of participants | 27 (13 postmenopausal women, 14 young women) | 31 (15 postmenopausal women, 16 young women) |  | In total, 58 (28 postmenopausal women, 30 young women) |
| Age | 30 to 60 years | 30 to 60 years |  | 28 postmenopausal women aged between 55 and 60 years, and 30 young women between 30 and 35 years. |
| Sex | Female | Female |  |  |
| Skin areas | Upper leg skin | Upper leg skin |  |  |
| Severity of dry skin | Normal skin hydration levels (clinical score 0 to1) | Dry-skin phenotype  (score 3 to 4) |  |  |
| **Molecular markers** | | | Quan. units |  |
| Corneodesmosin (metric not reported) | 1555 | 3560 | Arbitrary unit | Quantity extracted from graph.  Amount is increased with xerosis (129%) |
|  | p < 0.001 | |  |  |
| Annexin A2 (metric not reported) | 142 | 323 |  | Quantity extracted from graph.  Amount is increased with xerosis (127%) |
|  | p = 0.006 | |  |  |
| phosphatidylethanolamine-binding protein 1 (PEBP1) (metric not reported) | 375 | 915 |  | Quantity extracted from graph.  Amount is increased with xerosis (144%) |
|  | p = 0.002 | |  |  |

**Ishikawa et. al., 2013**

| Author | Ishikawa et. al. [35]  (Title: Dry skin in the winter is related to the ceramide profile in the stratum corneum and can be improved by treatment with a Eucalyptus extract) |
| --- | --- |
| Year | 2013 |
| Study design | Controlled clinical trial |
| Country/ ethnicity | United States of America (ethnicity: not reported) |
| Signs of dry skin and scoring method | Visual dryness  0= Normal skin – no signs of dryness,  2= Mild dryness – slight, but definite roughness; fine scaling present; may have a powdery or ashy appearance,  4= Moderate dryness – moderate roughness; somewhat coarser scaling; some cracking as evidenced by uplifted scales,  6= Marked dryness – marked roughness, coarse scaling; cracking evident as uplifted scales; some thickening may be present,  8= Severe dryness – verify marked roughness; very coarse scaling; cracking progressing to fissuring; erythema may be present; marked thickening may be present.  Tactile roughness  0= normal –smooth soft supple (yield without wrinkling) resilient  2= mild roughness –papery/parchment like feel; slight wrinkling upon manipulation  4= moderate roughness –slight sandy/grainy feel; skin wrinkles upon manipulation  6= marked roughness–coarse, rigid feel; somewhat brittle  8= severe roughness–rough feel, brittle; inflexible upon manipulation |
| Analysed material | Stratum corneum |
| Sampling technique | Skin surface sampling using D-Squame discs; lipid sampling by tape-stripping |
| Method of analysis | Liquid chromatography mass spectrometry |

|  | Test moisturizer (containing extract of *Eucalyptus globulus*) | | Control moisturizer | |  | Comments |
| --- | --- | --- | --- | --- | --- | --- |
| Number of participants | 20 female patients with dry skin | | Same participants | |  |  |
| Age  (mean, SD) | 47 years.  Age range: 32 to 57 years | | Same participants | |  |  |
| Sex | Female | | Same participants | |  |  |
| Skin areas | Outer calf of one leg | | Outer calf of the other leg | |  |  |
|  | Day 0 | | Day 28 | |  |  |
| Severity of dry skin | Moderate to severe (visual dryness score >4) | not reported | Moderate to severe (visual dryness score >4) | not reported |  |  |
| Visual Dryness, mean difference from Day 0 |  | -4.81 |  | -4.83 |  |  |
|  | p < 0.001 | | p < 0.001 | |  |  |
| **Molecular markers** | | | | | Quan. units |  |
|  | Day 0 | Day 28 | Day 0 | Day 28 |  |  |
| Ceramide [NP] (mean, SD) | 3.1 | 3.5  Mean difference from day 0 is 0.42 | 3.1 | 3.4  Mean difference from day 0 is 0.35 | μg/mg protein | Quantity extracted from graph. |
|  | p < 0.05 | | p < 0.05 | |  |  |
| Total Ceramide, mean difference from day 0 |  | 0.79 |  | -0.09 |  |  |
| Correlation between Ceramide levels and dryness | Day 0 (based on the average of both calves) | | | |  | Quantity of the analysed markers were not reported. |
| Ceramide [NP] (metric not reported) | r = -0.501 (p < 0.05) | | | |  |  |
| Total ceramide (metric not reported) | r = -0.471, (p < 0.05) | | | |  |  |
| Ceramide [NH] (metric not reported) | r = -0.445, (p < 0.05) | | | |  |  |
| Ceramide [NS] (metric not reported) | r = -0.433 | | | |  |  |
| Ceramide [NDS] (metric not reported) | r = -0.429 | | | |  |  |
| Ceramide [EOS] (metric not reported) | r = -0.401 | | | |  |  |
| Ceramide [AH] (metric not reported) | r = -0.389 | | | |  |  |
| Ceramide [EOH] (metric not reported) | r = -0.380 | | | |  |  |
| Ceramide [AS] (metric not reported) | r = -0.376 | | | |  |  |
| Ceramide [EOP] (metric not reported) | r = -0.361 | | | |  |  |
| Ceramide [ADS] (metric not reported) | r = -0.274 | | | |  |  |
| Ceramide [AP] (metric not reported) | r = -0.239 | | | |  |  |

**Schweiger et. al., 2013**

| Author | Schweiger et. al. [36]  (Title: efficacy of a new tonic containing urea, lactate, polidocanol, and *Glycyrrhiza inflata* root extract in the treatment of a dry, itchy, and subclinically inflamed scalp) |
| --- | --- |
| Year | 2013 |
| Study design | Randomized controlled trial, split-body comparison |
| Country/ ethnicity | Germany (ethnicity: not reported) |
| Signs of dry skin and scoring method | The various symptoms, scalp itching, tautness, and oiliness were determined based on the following assessment scale:  0 = no characteristic symptom;  1 = weak, even visible/perceivable symptom;  2 = mild symptom;  3 = moderate symptom;  4 = strong symptom;  5 = very strong (severe) symptom.  The approval rates (%) for following statements were determined using a self-assessment questionnaire:  A: scalp condition was improved perceivably  B: regular use reduces scalp dryness  C: regular use diminishes scalp itching  D: regular use perceivably reduces scalp tautness |
| Analysed material | For urea and lactate: Compounds dissolved from stratum corneum.  For free fatty acids, triglycerides, amide band ratio (I/II): direct analysis from scalp site  For cytokines: compounds dissolved from stratum corneum. |
| Sampling technique | For Urea and Lactate: The DIP-it sampler was rubbed 10 times with the enclosed end of the glass capillaries, while applying slight, constant pressure. The adherent skin surface material was directly analyzed without further sample preparation.  For free fatty acids, triglycerides, amide band ratio (I/II): to record a spectrum, the volunteer’s hair was parted; a N_2_ -cooled diamond measuring head was placed vertically on the test site. Five measurements consisting of 40 scans were carried out.  For cytokines: prewetted cotton buds was rubbed against the skin. |
| Method of analysis | For Urea and Lactate: direct analysis in real-time mass spectrometry (DART-MS).  For free fatty acids, triglycerides, hydration: fourier-transformed middle-infrared spectroscopy (FTMIR)  For cytokines: enzyme-linked immunosorbent assays (ELISA) |

|  |  | | |  | | |  | Comments |
| --- | --- | --- | --- | --- | --- | --- | --- | --- |
|  | **Untreated** | | | **Tonic treated** | | |  | Test tonic: aqueous tonic containing licochalcone A as main active ingredient. |
| Number of participants | 30 volunteers with dry and itchy scalp skin. | | | Same participants | | |  | 8 of the 21 participants (38%) reported a previous history of AD |
| Age | 26 to 73 years. | | | Same participants | | |  |  |
| Sex | 17 women, 13 men | | | Same participants | | |  |  |
| Skin areas | The one side of the scalp | | | The other side of the scalp | | |  |  |
|  | Baseline (t_0_) | After 2 weeks (t_1 untreated_) | After 4 weeks (t_2 untreated_) | Baseline (t_0_) | After 2 weeks of treatment (t_1 tonic treated_) | After 4 weeks of treatment (t_2 tonic treated_) |  |  |
| Severity of dry skin | Skin conductivity: <20 μS; Scalp oiliness score: <2.5 (by expert visual asessment);  Scalp itching and/or tautness score: ≥ 2 (by self-assessment of volunteers) | Not reported | Not reported | Skin conductivity: <20 μS; Scalp oiliness score: <2.5 (by expert visual asessment);  Scalp itching and/or tautness score: ≥ 2 (by self-assessment of volunteers) | 72% Volunteers (of 25) reported reduction in their scalp dryness by self-assessment. | 88% Volunteers (of 24) reported reduction in their scalp dryness by self-assessment. |  |  |
| **Molecular markers** | | | | | | | Quan. units |  |
| Amide band ratio I/II (metric not reported) | 100.3 | 105.5 | 99.0 | 100.3 | 113.2 | 105.0 | Percent relative to baseline | Amide band ratio I/II was measured as a second method for assessing the scalp moisturization.  Quantity extracted from graph. |
|  |  |  |  | p ≤ 0.05 | | |  |  |
| Urea (n=29); median | 102.8 | 99.6 | 80.1 | 102.8 | 264.1  p ≤ 0.05 compared to baseline and t_1 untreated_ | 160.2  p ≤ 0.05 compared to baseline, t_1 tonic treated_ and t_2 untreated_ | Percent relative to baseline | Quantity extracted from graph. |
| Lactate (n=29); median | 103.2 | 80.4 | 81.4 | 103.2 | 223.5  p ≤ 0.05 compared to baseline and t_1 untreated_ | 124.5  p ≤ 0.05 compared to baseline, t_1 tonic treated_ and t_2 untreated_ | Percent relative to baseline | Quantity extracted from graph. |
| Triglyceride (n=30); median | 100.2 | 91.1 | 80.9 | 100.2 | 112.4  p ≤ 0.05 compared to baseline and t_1 untreated_ | 114.7 | Percent relative to baseline | Quantity extracted from graph. |
| Free fatty acid (median) | 99.2 | 126.2 | 108.7 | 99.2 | 65.9  p ≤ 0.05 compared to baseline and t_1 untreated_ | 57.1  p ≤ 0.05 compared to baseline, t_1 tonic treated_ andt_2 untreated_ | Percent relative to baseline | Quantity extracted from graph. |
| Total lipid (median) | 100.3 | 105.4 | 97.1 | 100.3 | 115.9 | 109.6 | Percent relative to baseline | Quantity extracted from graph. |
|  | p value not reported | | | | | |  |  |
| IL-1ra/IL-1β (median) | 101.2 | 102.5 | 95.0 | 101.2 | 78.1  p ≤ 0.05 compared to baseline and t_1 untreated_ | 76.1  p≤0.05 compared to t_2 untreated_ | Percent relative to baseline | Quantity extracted from graph. |
| IL-8 (median) | 99.6 | 67.4 | 74.0 | 99.6 | 38.3 | 55.1 | Percent relative to baseline | Quantity extracted from graph. |
|  |  |  |  | p ≤ 0.05 | |  |  |  |

**Son et. al., 2015**

| Author | Son et. al. [37]  (Title: Skin dryness in apparently healthy human skin is associated with decreased expression of bleomycin hydrolase in the stratum corneum) |
| --- | --- |
| Year | 2015 |
| Study design | Cross sectional study, split-body comparison |
| Country/ ethnicity | Korea (ethnicity: Asian) |
| Signs of dry skin and scoring method | Not reported  (The capacitance values for hydrated skin and for dry skin were > 29 AU and < 25 AU, respectively. The most hydrated area of the volunteer’s right forearm was classified as ‘hydrated skin’ and the least hydrated area of the left forearm of the same volunteer was classified as ‘dry skin’. |
| Analysed material | Stratum corneum. |
| Sampling technique | Tapes were attached to the volar forearm skin using a disc pressure applicator, and five sequential tape strippings with five different tapes were performed on each hydrated and dry skin region. |
| Method of analysis | For natural moisturizing factors: high performance liquid chromatography,  For (pro)filaggrin and proteases: western blotting and densitometric analyses. |

|  | Hydrated skin | Dry skin | Quan. units | Comments |
| --- | --- | --- | --- | --- |
| Number of participants | 22 (15 provided samples) | Same participants |  |  |
| Age (Mean, SD) | Men: 33.8 (5.6) years  Women: 31.3 (4.1) years | Same participants |  | Age is presented for the participants who provided samples |
| Sex | 11 men (8 provided samples),  11 women (7 provided samples) | Same participants |  |  |
| Skin areas | The volar forearm (right) | The volar forearm (left) |  |  |
| Severity of dry skin | capacitance value > 29 AU | capacitance value < 25 AU | Arbitrary unit |  |
| **Molecular markers** | | | | |
| (Pro)filaggrin expression (Average) | 100.0 | 101.4 | percent of ratio | Quantity extracted from graph |
|  | p value not reported | |  |  |
| Relative bleomycin hydrolase expression (Average) | 106.0 | 86.2 | Percent |  |
|  | p < 0.05 | |  |  |
| Total NMFs (as free amino acid) (Average) | 173.6 | 143.4 | µg/ mg SC proteins |  |
|  | p < 0.05 | |  |  |
| Histidine (Average) | 12.0 | 9.3 |  |  |
|  | p < 0.05 | |  |  |
| Serine (Average) | 43.0 | 35.0 |  |  |
|  | p < 0.05 | |  |  |
| Arginine (Average) | 6.8 | 4.8 |  |  |
|  | p value not reported | |  |  |
| Glycine (Average) | 14.8 | 12.3 |  |  |
|  | p < 0.05 | |  |  |
| Aspartic acid (Average) | 8.3 | 7.6 |  |  |
|  | p value not reported | |  |  |
| Glutamic acid (Average) | 35.3 | 28.0 |  |  |
|  | p < 0.05 | |  |  |
| Threonine (Average) | 11.3 | 9.0 |  |  |
|  | p value not reported | |  |  |
| Alanine (Average) | 15.5 | 13.3 |  |  |
|  | p value not reported | |  |  |
| gamma-Aminobutyric acid (Average) | 8.5 | 8.0 |  |  |
|  | p value not reported | |  |  |
| Proline (Average) | 4.0 | 3.5 |  |  |
|  | p value not reported | |  |  |
| Lysine (Average) | 3.5 | 3.5 |  |  |
|  | p value not reported | |  |  |
| Tyrosine (Average) | 3.5 | 2.8 |  |  |
|  | p value not reported | |  |  |
| Methionine (Average) | 0.8 | 0.4 |  |  |
|  | p value not reported | |  |  |
| Valine (Average) | 4.5 | 4.0 |  |  |
|  | p value not reported | |  |  |
| Leucine (Average) | 3.0 | 2.3 |  |  |
|  | p < 0.05 | |  |  |
| Isoleucine (Average) | 2.5 | 2.3 |  |  |
|  | p value not reported | |  |  |
| Phenylalanine (Average) | 2.0 | 1.5 |  |  |
|  | p < 0.05 | |  |  |
| Tryptophan (Average) | 2.0 | 1.3 |  |  |
|  | p value not reported | |  |  |
| Pyrrolidone carboxylic acid (Average) | 7.0 | 6.0 |  |  |
|  | p value not reported | |  |  |
| Urocanic acid (Average) | 2.0 | 1.8 |  |  |
|  | p value not reported | |  |  |

**Danby et. al., 2016**

| Author | Danby et. al. [38]  (Title: The effect of an emollient containing urea, ceramide NP, and lactate on skin barrier structure and function in older people with dry skin) |
| --- | --- |
| Year | 2016 |
| Study design | Randomized controlled clinical trial (intra-individual comparison) |
| Country/ ethnicity | United Kingdom (ethnicity: not reported) |
| Signs of dry skin and scoring method | Skin dryness on a 5-point scale.  1 = no dryness.  5= severe dryness with cracking and lifting scales. |
| Analysed material | For stratum corneum protease activity and PCA: stratum corneum,  For Lactate: compounds dissolved from stratum corneum |
| Sampling technique | Tape-stripping (strips 4–6 pooled),  Prewetted cotton swab was rubbed against the skin and then transferred to 1 ml PBS. |
| Method of analysis | For stratum corneum protease activity: caseinolytic, chymotrypsin-like and trypsin-like activities were determined using corresponding substrates.  For PCA: it was referred to a previous publication, however no statement of any analytical procedure was found there.  For lactate: fluorometric L -lactate assay.  For carboxylic acid levels : fourier transform infrared spectroscopy |

|  | | | | |  | Comments  Test emollient: lactate |
| --- | --- | --- | --- | --- | --- | --- |
| **Cohort 1** | Test emollient | | No treatment | |  |  |
| Number of participants | 21 volunteers with dry skin | | Same participants | |  | 8 of the 21 participants (38%) reported a previous history of AD |
| Age  (mean) | 69 years.  Age range: 60 to 89 years | | Same participants | |  |  |
| Sex | 17 women, 4 men | | Same participants | |  |  |
| Skin areas | One forearm (volar side, 3 cm below elbow flexure to 3 cm above the wrist) | | The other forearm | |  |  |
|  | Day 0 | After 28 days | Day 0 | After 28 days |  |  |
| Severity of dry skin | Mean score 3 | Not reported | Mean score 3 | Not reported |  | It was reported that the test emollient hydrated the skin. |
| **Molecular markers** | | | | | Quan. units |  |
| Caseinolytic activities (metric not reported) | Not reported | 0.86 | Not reported | 1.48 | nU/μg | Quantity extracted from graph. |
|  |  | p = 0.0023 | | |  |  |
| Chymotrypsin-like activities (metric not reported) | Not reported | 1.09 | Not reported | 2.68 | nU/μg | Quantity extracted from graph. |
|  |  | p < 0.0001 | | |  |  |
| Trypsin-like activities after (metric not reported) | Not reported | 1.71 | Not reported | 2.62 | nU/μg | Quantity extracted from graph. |
|  |  | p value not reported | | |  |  |

| **Cohort 3** | **Test emollient** | | **Control emollient** | |  |  |
| --- | --- | --- | --- | --- | --- | --- |
| Number of participants | 21 volunteers with dry skin; 18 completed the study. | | Same participants | |  | 6 of the 18 participants (33%) reported a previous history of AD |
| Age  (mean, SD) | 68 years.  Age range: 60 to 79 years | | Same participants | |  |  |
| Gender | 14 women, 7 men | | Same participants | |  |  |
| Skin areas | One forearm (volar side, 3 cm below elbow flexure to 3 cm above the wrist) | | Another forearm (volar side, 3 cm below elbow flexure to 3 cm above the wrist) | |  |  |
|  | Day 0 | After 28 days | Day 0 | After 28 days |  |  |
| Severity of dry skin | Mean score 3 | Not reported | Mean score 3 | Not reported |  |  |
| **Molecular markers** | | | | | | |
| Caseinolytic activities (metric not reported) | Not reported | 1.20 | Not reported | 1.54 | nU/μg | Quantity extracted from graph. |
|  |  | p < 0.05 | | |  |  |
| Chymotrypsin-like activities (metric not reported) | Not reported | 1.36 | Not reported | 1.54 |  |  |
|  |  | p value not reported | | |  |  |
| Trypsin-like activities (metric not reported) | Not reported | 2.82 | Not reported | 4.00 |  |  |
|  |  | p < 0.05 | | |  |  |
| Lactate (metric not reported) | 406.7 | 690.0 | 383.3 | 350.0 | nmol/sample |  |
|  |  | p < 0.05 | | |  |  |
| Pyrrolidone carboxylic acid (PCA) (metric not reported) | 513.3 | 733.3 | 559.9 | 600.0 | μmol/g protein |  |
|  | p < 0.05 | |  |  |  |  |
|  |  | p = 0.0002 | | |  |  |
| Carboxylicacidlevels (metric not reported) | 0.32 | 0.40 | 0.32 | 0.31 | Absorbance | Quantity extracted from graph.  FTIR-determination based on absorbance at 1,410 cm^–1^ /amide II. |
|  | p < 0.05 | |  |  |  |  |
|  |  | p ≤ 0.0001 | | |  |  |

**Tamura et. al., 2016**

| Author | Tamura et. al. [39]  (Title: The roughness of lip skin is related to the ceramide profile in the stratum corneum) |
| --- | --- |
| Year | 2016 |
| Study design | Cross sectional study |
| Country/ ethnicity | Japan (ethnicity: Asian) |
| Signs of dry skin and scoring method | The degree of lip roughness in each subject was classified according to the criteria described below:  Score 0: no desquamation,  Score 1: slightly desquamated,  Score 2: heavily desquamated |
| Analysed material | Stratum corneum received from each lip side |
| Sampling technique | Tape stripping with polyphenylsulphide film tape. |
| Method of analysis | Liquid chromatography mass spectrometry. |

|  | Subjects having no desquamation on lips | Subjects having slightly desquamated lips | Subjects having slightly desquamated lips | Quan. units | Comments |
| --- | --- | --- | --- | --- | --- |
| Number of participants | 41 | | |  | Data regarding the distribution of participants in different groups was not provided |
| Age (mean) | 34.1 years  Age range: 22 to 52 years | | |  |  |
| Sex | Female | | |  |  |
| Skin areas | Lips | | |  |  |
| Severity of dry skin | Score 0 | Score 1 | Score 2 |  |  |
| **Molecular markers** | | | | | |
| Ceramide (NH) (metric not reported) | 2.5 | 2.3 | 1.9 | µg/mg |  |
|  | r = -0.371 (p < 0.05) | | |  |  |
| Ceramide (NP) (metric not reported) | 1.6 | 1.4 | 1.2 |  |  |
|  | r = -0.420 (p < 0.01) | | |  |  |

**Vyumvuhoreet. al., 2018**

| Author | Vyumvuhore et. al., [40]  (Title: Lipid organization in xerosis: the key of the problem?) |
| --- | --- |
| Year | 2018 |
| Study design | Cross sectional study |
| Country/ ethnicity | France (ethnicity: not reported) |
| Signs of dry skin and scoring method | The dryness was visually evaluated according to a scale scoring from 1 to 4 as previously described in Byrne 2010.  Grade 1: healthy skin, no visible signs of dryness and a healthy sheen and glow.  Grade 2: indicates mild xerosis, characterized by small ﬂakes of dry skin and whitening of dermatoglyphic triangles.  Grade 3: moderate xerosis; appearance of small, dry ﬂakes causing a powdery appearance. Corners of the dermatoglyphic triangles start to uplift.  Grade 4: well-deﬁned xerosis with the entire length of a number of dermatoglyphic triangles uplifted to generate large, dry ﬂakes. Roughness and redness are readily apparent. |
| Analysed material | Compounds dissolved from stratum corneum. |
| Sampling technique | Cotton swabs wetted with extraction agent. |
| Method of analysis | Liquid chromatography mass spectrometry. |

|  | Subjects with normal skin | Subjects with mild xerosis |  | Comments |
| --- | --- | --- | --- | --- |
| Number of participants | 15 | 19 |  |  |
| Age (mean) | 58 years | 57 years |  |  |
| Sex | Not mentioned | Not mentioned |  |  |
| Skin areas | On outside arms or the calf | On outside arms or the calf |  |  |
| Severity of dry skin | Grade 1 | Grade 3 to 4 |  |  |
| **Molecular markers** | | | Quan. units |  |
| C_65_H_126_NO_6;_ presumably, ceramide (NdS) (metric not reported) | 11801 | 1985 | Intensity (Arbitrary unit) | Quantity extracted from graph |
|  | P < 0.05 | |  |  |
| C_66_H_128_NO_6;_ presumably, ceramide (NS) (metric not reported) | 4595 | 1191 |  |  |
|  | p value not reported | |  |  |
| C_67_H_130_NO_6;_ presumably, ceramide (EOP) (metric not reported) | 6695 | 1305 |  |  |
|  | P < 0.05 | |  |  |

**Lechner et. al., 2019**

| Author | Lechner et al. [41]  (Title: Comparing skin characteristics and molecular markers of xerotic foot skin between diabetic and non-diabetic subjects: an exploratory study) |
| --- | --- |
| Year | 2019 |
| Study design | Cross sectional |
| Country/ ethnicity | Germany (ethnicity: not reported) |
| Signs of dry skin and scoring method | 0 = Normal skin; no sign of dryness.  1= Dusty appearance.  2= Presence of many particles of minute skin flakes.  3= Defined (usually circular) scaling.  4= Well-defined scaling with larger raised edges Size.  5= Large-scale plates  6= Large-scale plates with high lifting of scale edges. Deep erythematous fissures between scale plates.  Moderate dryness: Met the criteria of grades 3 and 4 in regard of scaling and/or showed only superficial fissures limited to the epidermis.  Severe dryness: Met the criteria of grade 5 in regard of scaling and/or showed deep heel fissures extending to dermis.  (Please see Rogers et al., 1989 and Oe et al., 2012) |
| Analysed material | Compounds dissolved from stratum corneum |
| Sampling technique | Cotton swabs wetted with chelating agents and non ionic surfactants |
| Method of analysis | Liquid chromatography mass spectrometry |

|  | Non-diabetic xerosis | | Diabetic xerosis | |  | Comments |
| --- | --- | --- | --- | --- | --- | --- |
| Number of participants | n = 20 (Samples collected from: 15) | | n = 40 (Samples collected from: 30) | |  |  |
| Age  (mean, SD) | 56.2 (9.3) | | 63.5 (7.8) | |  |  |
| Sex | Females =15, Males =5 | | Females =13, Males =27 | |  |  |
| Skin areas | Foot dorsum | Plantar heel | Plantar heel | Foot dorsum |  |  |
| Severity of dry skin | Moderate = 7, Severe = 13 | Moderate = 7, Severe = 13 | Moderate = 20, Severe = 20 | Moderate = 20, Severe = 20 |  |  |
| **Molecular markers** | | | | | Quan. units |  |
| Ceramides (mean, SD) | 95.1 (35.4) | 430.4 (97.1) | 824.5 (550.7) | 283.6 (146.2) | UA/cm^2^ | Amount is increased in diabetics |
|  |  | p = 0.003 | |  |  |  |
|  | p < 0.001 | | | |  |  |
| NMFs (mean, SD) | 65.0 (37.1) | 148.4 (86.0) | 199.0 (113.2) | 101.7 (70.4) | μg/cm^2^ |  |
|  | p value not reported | | | |  |  |
| Amino Acid (mean, SD) | 39.8 (12.7) | 90.9 (42.2) | 139.0 (67.4) | 67.5 (40.1) | UA/cm^2^ | Amount is increased in diabetics |
|  |  | p = 0.01 | |  |  |  |
|  | p = 0.02 | | | |  |  |
| Serine (mean, SD) | 42.4 (25.5) | 99.1 (60.6) | 145.8 (72.5) | 67.3 (41.2) | μg/cm^2^ | Amount is increased in diabetics |
|  |  | p = 0.02 | |  |  |  |
|  | p = 0.04 | | | |  |  |
| Pyrrolidone carboxylic acid (mean, SD) | 54.1 (31.2) | 125.5 (75.4) | 172.3 (96.8) | 86.1 (57.4) | μg/cm^2^ | Amount is increased in diabetics |
|  | p value not reported | | | |  |  |
| Urocanic acid trans (mean, SD) | 6.0 (4.6) | 16.4 (9.7) | 20.2 (13.2) | 10.3 (10.0) | μg/cm^2^ | Amount is increased in diabetics |
|  | p = 0.03 | | | |  |  |
| Urocanic acid cis (mean, SD) | 4.9 (2.6) | 6.5 (5.5) | 6.5 (5.8) | 5.3 (4.7) | μg/cm^2^ | Amount is increased in diabetics |
|  | p value not reported | | | |  |  |
| Histamine (mean, SD) | 5.3 (2.9) | 9.0 (5.2) | 23.3 (15.0) | 13.5 (11.5) | ng/cm^2^ | Amount is increased in diabetics |
|  |  | p < 0.001 | |  |  |  |
|  | p = 0.005 | | | |  |  |
| Total proteins (mean, SD) | 28.7 (15.2) | 66.5 (56.2) | 101.5 (43.8) | 42.2 (17.7) | μg/ml | Amount is increased in diabetics |
|  |  | p = 0.003 | |  |  |  |
|  | p = 0.02 | | | |  |  |
| Glutathione (mean, SD) | 31.5 (8.0) | 35.9 (9.4) | Not detected | Not detected | ng/cm^2^ | Not detected in diabetics |
| Melondialdehyde (mean, SD) | 60.8 (7.2) | 66.7 (12.5) | 58.7 (14.9) | 47.7 (8.6) | ng/cm^2^ | Amount is decreased in diabetics |
|  |  | p = 0.03 | |  |  |  |
|  | p < 0.001 | | | |  |  |

**Legiawati et al 2020**

| Author | Legiawati et. al. [42]  (Title: Oral and topical *Centella asiatica* in type 2 diabetes mellitus patients with dry skin: a three-arm prospective randomized double-blind controlled trial) |
| --- | --- |
| Year | 2020 |
| Study design | Randomized controlled trial |
| Country/ ethnicity | Indonesia (ethnicity: Asian) |
| Signs of dry skin and scoring method | The status of skin dryness was assessed by specified symptom sum score (SRRC) system with grading of scaling, roughness, redness and cracks as the main signs of dry skin (xerosis).  Scaling (visual evaluation)  *0 = absent*  1 *= slight;* Small scales only, surface lightly dull in colour,  *2=moderate;* Small scales in combination with larger scales (>0.05 mm), surface opaque or whitish,  *3 =severe;* Larger and large scales (flakes >1 mm) are prominent, surface whitish  4 *=extreme;* Larger flakes covering almost the entire skin surface in the examination field  Roughness (tactile evaluation)  *0 =absent;* Perfectly smooth and pliable  1 *=slight;* Slightly irregular and scratchy on tangential tactile evaluation  *2=moderate;* Definitely irregular and scratchy and possibly slightly stiffened on vertical tactile evaluation  *3 =severe;* Advanced irregularly and scratchy feeling associated with some stiffening.  4 *=extreme;*Gross irregularity and major disturbance of skin markings and definite stiffening.  Redness (visual)  0 *=absent*  1 *=slight;* Small areas of minimal redness or diffuse faint redness  *2=moderate;* Limited areas of definite redness or diffuse and obvious redness  3 = *severe;*Larger areas of definite redness or diffuse and more pronounced redness.  *4 =extreme;* Advanced redness in entire examination field (redness of cracks not included)  Cracks fissures (visual evaluation)  *O=absent*  1 = *slight;* Single and superficial cracks in the examinationfield  *2=moderate;* Single or grouped superficial and more deep cracks  3*=severe;* As *2* but with deep cracks  *4=extreme;*Dominated by deep cracks.  (Serup et al 1995) |
| Analysed material | Stratum corneum |
| Sampling technique | Cyanoacrylate skin surface stripping using a transparent foil of 3.75 cm × 2.5 cm with 20 μL cyanoacrylate adhesive. |
| Method of analysis | Enzyme-linked immunosorbent assays (ELISA) |

|  |  | | |  | | |  | | |  | Comments |
| --- | --- | --- | --- | --- | --- | --- | --- | --- | --- | --- | --- |
|  | Oral treatment and Topical treatment (CAo andCAt) | | | Oral placebo and Topical treatment (Plo andCAt) | | | Oral placebo and Topical placebo (Plo andPlt) | | |  | CAo= Oral dose of *Centellaasiatica* (2 ×1100 mg)  CAt= 1% ointment of *Centellaasiatica*  Plt= Vaseline album |
| Number of participants | 53 T2DM patients with dry skin on low extremities (SRRC score above 3) | | | 53 T2DM patients with dry skin on low extremities (SRRC score above 3) | | | 53 T2DM patients with dry skin on low extremities (SRRC score above 3) | | |  | 43, 42, 36, respectively, reported previous histories of atopy |
| Age (median, min to max) | 52 (34 to 58) | | | 54 (26 to 59) | | | 53 (34 to 59) | | |  |  |
| Sex | 13 Males, 40 Females | | | 14 Males, 39 Females | | | 12 Males, 41 Females | | |  |  |
| Skin areas | Right lower extremities | | | Right lower extremities | | | Right lower extremities | | |  |  |
|  | Day 1 | Day 15 | Day 29 | Day 1 | Day 15 | Day 29 | Day 1 | Day 15 | Day 29 |  |  |
| Severity of dry skin (SRRC); median (min to max) | 4  (3 to 10) | 2  (0 to 6) | 2  (0 to 6) | 4  (3 to 8) | 3  (0 to 7) | 2  (0 to 7) | 5  (3 to 8) | 3  (0 to 7) | 2  (0 to 6) |  |  |
| **Molecular markers** | | | | | | | | | | Quan. units |  |
| N(6)-carboxymethyl-lysine activity; median (min to max) | 87.2 (20.12 to 14559.42) | Not analyzed | 119.8 (24.2 to 615.9) | 77.2 (4.1 to 385.7) | Not analyzed | 119.4 (25.2 to 1731.4) | 93.9 (14 to 407.8) | Not analyzed | 104.8 (22 to 748.1) | pg/mg protein |  |
|  | p = 0.76 (on day 1), p = 0.41 (on day 29) | | | | | | | | |  |  |
| Interleukin-1α activity; median (min to max) | 16.5 (2.9 to 167.3) | Not analyzed | 19.7 (3.2 to 167.3) | 16.0 (2.1 to 110.5) | Not analyzed | 18.2 (4.9 to 69.6) | 17.6 (4.4 to 114.6) | Not analyzed | 17.6 (4.9 to 114.5) |  |  |
|  | p = 0.60 (on day 1), p = 0.68 (on day 29) | | | | | | | | |  |  |
| Superoxide dismutase activity; median (min to max) | 4.6 (0.3 to 59.4) | Not analyzed | 5.9 (1 to 59.4) | 3.4 (0.3 to 41.5) | Not analyzed | 4.3 (0.2 to 18.7) | 3.9 (0.2 to 35) | Not analyzed | 4.9 (0.1 to 23.6) | U/mg protein |  |
|  | p = 0.31 (on day 1), p = 0.07 (on day 29) | | | | | | | | |  |  |

| **Subgroup Analysis in partially controlled blood glucose subgroup:** | | | | | | | | | | | |
| --- | --- | --- | --- | --- | --- | --- | --- | --- | --- | --- | --- |
|  | Cao and CAt | | | Plo and CAt | | | Plo and Plt | | |  |  |
|  | Day 1  n=13 | Day 15 | Day 29  n=13 | Day 1  n=9 | Day 15 | Day 29  n=7 | Day 1  n=14 | Day 15 | Day 29  n=14 |  |  |
| Severity of dry skin (SRRC value) | 4 | 2 | 3 | Not reported | Not reported | Not reported | Not reported | Not reported | Not reported |  |  |
| **Molecular markers** | | | | | | | | | | | |
| N(6)-carboxymethyl-lysine activity; median (min to max) | 73.9 (26.7 to 219.9) | Not analyzed | 158 (26 to 524.6) | 153 (35.5 to 179.6) | Not analyzed | 82.7 (44.3 to 270.1) | 70.9 (27.5 to 279.4) | Not analyzed | 91.8 (22 to 422.1) | pg/mg protein |  |
|  | p = 0.55 (on day 1), p = 0.42 (on day 29) | | | | | | | | |  |  |
| Interleukin-1α activity; median (min to max) | 17.3 (7 to 32) | Not analyzed | 21.6 (7.9 to 65.9) | 17.9 (4.4 to 96.2) | Not analyzed | 17 (6.2 to 32.2) | 16.7 (6.8 to 47.1) | Not analyzed | 14.5 (4.9- to 80) |  |  |
|  | p = 0.67 (on day 1), p = 0.51 (on day 29) | | | | | | | | |  |  |
| Superoxide dismutase activity; median (min to max) | 3.9 (0.3 to 11.2) | Not analyzed | 8.4 (1.3 to 25) | 6 (0.4 to 25.3) | Not analyzed | 2.4 (1.3 to 10.5) | 2.7 (0.2 to 16.4) | Not analyzed | 3.5 (0.2 to 8.1) | U/mg protein |  |
|  | p = 0.28 (on day 1), **p = 0.03** (on day 29) | | | | | | | | |  |  |

| **Subgroup Analysis in well controlled, partially controlled and poorly controlled blood glucose subjects of the CAo+CAt treatment group:** | | | | | | | | | | | |
| --- | --- | --- | --- | --- | --- | --- | --- | --- | --- | --- | --- |
|  | well controlled | | | partially controlled * | | | poorly controlled | | |  |  |
|  | Day 1 | Day 15 | Day 29 | Day 1 | Day 15 | Day 29 | Day 1 | Day 15 | Day 29 |  |  |
| Severity of dry skin (SRRC value) | 4 | 2 | 1 | 4 | 2 | 3 | 4 | 3 | 3 |  | -Quantity extracted from graph. |
| **Molecular markers** | | | | | | | | | | | |
| N(6)-carboxymethyl-lysine activity (metric not reported) | 103.7 | Not analyzed | 125.9 | 71.6 | Not analyzed | 153.0 | 76.5 | Not analyzed | 118.5 | pg/mg protein | -Quantity extracted from graph. |
|  | p value not reported | | | | | | | | |  |  |
| Interleukin-1α activity (metric not reported) | 18.5 | Not analyzed | 22.0 | 17.2 | Not analyzed | 22.0 | 16.6 | Not analyzed | 14.3 |  | -Quantity extracted from graph. |
|  | p value not reported | | | | | | | | |  |  |
| Superoxide dismutase activity (metric not reported) | 4.6 | Not analyzed | 6.6 | 4.1 | Not analyzed | 8.6 | 5.5 | Not analyzed | 5.3 | U/mg protein | -Quantity extracted from graph. |
|  | p value not reported | | | | | | | | |  |  |

*****These values differ a little bit from above, as they were extracted from the graphs.

**Uchino et. al., 2020**

| Author | Uchino et. al. [43]  (Title: Association of dry skin with intercellular lipid composition of stratum corneum after erlotinib administration) |
| --- | --- |
| Year | 2020 |
| Study design | Controlled clinical trial |
| Country/ ethnicity | Japan (ethnicity: not reported) |
| Signs of dry skin and scoring method | The condition of dry skin was assessed according to the ‘Common Terminology Criteria for Adverse Events (CT-CAE)’ version 4.0; term definition: a disorder characterized by flaky and dull skin; the pores are generally fine, the texture is a papery thin texture.  Grade 1: mild; asymptomatic or mild symptoms; covering <10% Body surface area and no associated erythema or pruritus,  Grade 2: moderate; covering 10 - 30% BSA and associated with erythema or pruritus; limiting instrumental activities of daily living (ADL)  Grade 3: severe or medically significant; covering > 30% BSA and associated with pruritus; limiting self-care ADL. |
| Analysed material | Stratum corneum. |
| Sampling technique | Tape-stripping. Each tape was pressed against the skin for 10 s using a standardized pressurizer to minimize the pressure associated with sampling. Each of the fifth tape-stripped tapes corresponding to each sampling time point was cut into half and used for extraction of compounds. |
| Method of analysis | For lipids: ultra performance liquid chromatography combined with time-of-flight mass spectrometry.  For proteins: ortho-phthalaldehyde (OPA) fluorescent protein assay. |

|  | Healthy Subjects | | | Patients with non-small lung cancer receiving oral erlotinib administration (150 mg/day) | | | | |  | Comments |
| --- | --- | --- | --- | --- | --- | --- | --- | --- | --- | --- |
| Number of participants | 6 | | | 18 | | | | |  |  |
| Age | 50-60 years | | | 62-85 years | | | | |  | Median= 74 |
| Sex | Not mentioned | | | 10 Males, 8 Females | | | | |  |  |
| Skin areas | Inner forearm | | | Inner forearm | | | | |  |  |
|  | Day 0 | Day 28 | Day 56 | Day 0 | Day 7 | Day 14 | Day 28 | Day 56 |  |  |
| Severity of dry skin | Not reported | Not reported | Not reported | Grade 0  = 66.5%,  grade 1  = 33.5% | Grade 0 = 94%,  grade 1 = 6% | Grade 0 = 43.6%,  grade 1 = 37.6%,  grade 2 = 6%,  grade 3 = 12.8% | Grade 0 = 16.3%,  grade 1 = 72.8%,  grade 2 = 10.9% | Grade 0  = 13.9%,  grade 1 = 21.5%  grade 2 = 64.6% | Percentage | Quantity extracted from graph.  Dry skin increased with increasing time after the initiation of erlotinib administration. |
| **Molecular markers** | | | | | | | | | Quan. units |  |
| Cholesterol sulfate (metric not reported) | 1.00 | 1.05 | 0.82 | 1.00 | 0.87 | 0.87 | 1.50 | 1.82 | Enhancement ratio | Quantity extracted from graph |
|  |  |  |  | **p < 0.05, between day 0 and day 56, day 7 and day 56, day 14 and day 56** | | | | |  |  |
| Total free fatty acid (metric not reported) | 0.062 | 0.066 | 0.062 | 0.077 | 0.076 | 0.054 | 0.050 | 0.041 | Ratio of free fatty acid abundance to protein concentration |  |
|  | p value not reported | | | | | | | |  |  |
| Saturated free fatty acid (metric not reported) | 0.009 | 0.008 | 0.009 | 0.018 | 0.018 | 0.013 | 0.012 | 0.010 |  |  |
|  | p value not reported | | | | | | | |  |  |
| Hydroxyfree fatty acid (metric not reported) | 0.052 | 0.056 | 0.052 | 0.059 | 0.058 | 0.040 | 0.036 | 0.030 |  |  |
|  |  |  |  | **p < 0.05, between day 0 and day 56, day 7 and day 56** | | | | |  |  |
| Unsaturated free fatty acid (metric not reported) | 0.00057 | 0.00063 | 0.00060 | 0.00079 | 0.00113 | 0.00066 | 0.00070 | 0.00080 |  |  |
|  | p value not reported | | | | | | | |  |  |
| Total ceramide (metric not reported) | 0.30 | 0.37 | 0.35 | 0.58 | 0.49 | 0.34 | 0.38 | 0.38 | Ratio of CER abundance to protein concentration |  |
|  | p value not reported | | | | | | | |  |  |
| Ceramide [NdS] (metric not reported) | 0.024 | 0.024 | 0.024 | 0.038 | 0.035 | 0.022 | 0.026 | 0.031 |  |  |
|  | p value not reported | | | | | | | |  |  |
| Ceramide [NS] (metric not reported) | 0.017 | 0.019 | 0.019 | 0.032 | 0.026 | 0.016 | 0.031 | 0.031 |  |  |
|  | p value not reported | | | | | | | |  |  |
| Ceramide [NP] (metric not reported) | 0.073 | 0.070 | 0.071 | 0.140 | 0.124 | 0.091 | 0.092 | 0.092 |  |  |
|  | p value not reported | | | | | | | |  |  |
| Ceramide [NH] (metric not reported) | 0.035 | 0.034 | 0.034 | 0.077 | 0.065 | 0.046 | 0.052 | 0.054 |  |  |
|  | p value not reported | | | | | | | |  |  |
| Ceramide [AdS] (metric not reported) | 0.005 | 0.005 | 0.005 | 0.013 | 0.010 | 0.010 | 0.013 | 0.012 |  |  |
|  | p value not reported | | | | | | | |  |  |
| Ceramide [AS] (metric not reported) | 0.013 | 0.015 | 0.015 | 0.029 | 0.022 | 0.014 | 0.030 | 0.022 |  |  |
|  | p value not reported | | | | | | | |  |  |
| Ceramide [AP] (metric not reported) | 0.052 | 0.052 | 0.048 | 0.107 | 0.084 | 0.066 | 0.066 | 0.052 |  |  |
|  |  |  |  | **p < 0.05, between day 0 and day 56** | | | | |  |  |
| Ceramide [AH] (metric not reported) | 0.051 | 0.050 | 0.048 | 0.092 | 0.076 | 0.054 | 0.061 | 0.056 |  |  |
|  |  |  |  | **p < 0.05, between day 0 and day 56, day 0 and day 14** | | | | |  |  |
| Ceramide [EOdS] (metric not reported) | 0.003 | 0.004 | 0.003 | 0.005 | 0.004 | 0.001 | 0.002 | 0.003 |  |  |
|  | p value not reported | | | | | | | |  |  |
| Ceramide [EOS] (metric not reported) | 0.011 | 0.012 | 0.012 | 0.019 | 0.013 | 0.008 | 0.009 | 0.011 |  |  |
|  | p value not reported | | | | | | | |  |  |
| Ceramide [EOP] (metric not reported) | 0.003 | 0.004 | 0.003 | 0.010 | 0.006 | 0.004 | 0.005 | 0.005 |  |  |
|  | p value not reported | | | | | | | |  |  |
| Ceramide [EOH] (metric not reported) | 0.011 | 0.012 | 0.012 | 0.023 | 0.019 | 0.011 | 0.013 | 0.015 |  |  |
|  | p value not reported | | | | | | | |  |  |

| **Reported only for patients after erlotinib administration:** | | | | | | | | | | | | |  |  |
| --- | --- | --- | --- | --- | --- | --- | --- | --- | --- | --- | --- | --- | --- | --- |
|  | Day 14 | | | | Day 28 | | | | Day 56 | | | |  |  |
| Dry skin grade | 0 | 1 | 2 | 3 | 0 | 1 | 2 | 3 | 0 | 1 | 2 | 3 |  |  |
| **Molecular markers** | | | | | | | | | | | | | | |
| Increase of cholesterol sulfate (mean) | 0.62 | 1.18 | 0.50 | 0.78 | 0.81 | 1.64 | 2.25 | Non-existent | 0.88 | 1.04 | 2.08 | non-existent | Ratio | Quantity extracted from graph |
|  | p value not reported | | | | | | | | | | | |  |  |
| Reduction of hydroxy free fatty acid (mean) | 0.65 | 0.89 | 0.52 | 0.92 | 0.60 | 0.71 | 0.86 | Non-existent | 0.62 | 0.52 | 0.70 | non-existent |  |  |
|  | p value not reported | | | | | | | | | | | |  |  |
| Reduction of ceramide (mean) | 0.63 | 0.78 | 0.52 | 0.78 | 0.65 | 0.75 | 1.18 | Non-existent | 0.68 | 0.52 | 0.96 | non-existent |  |  |
|  | p value not reported | | | | | | | | | | | |  |  |
