# Supplementary material for: Molecular characterization of xerosis cutis: A systematic review
Source: PLoS One. 2021 Dec 16;16(12):e0261253. doi: 10.1371/journal.pone.0261253 (PMC8675746; doi:10.1371/journal.pone.0261253)
Supplement: S3 Appendix — (DOCX) [file pone.0261253.s003.docx]

S3 Appendix: Molecular markers analyzed only once.

| Molecular markers | Analysed material | Sampling technique | Method of analysis | Association |
| --- | --- | --- | --- | --- |
| Aluminium level in the epidermis and dermis [23] | Epidermis and dermis | Separation of the epidermis from the dermis | Atomic absorption spectrophotometry | Yes |
| Saturated free fatty acids [43] | Stratum corneum | Tape stripping | Liquid chromatography mass spectrometry | Unclear |
| Hydroxy free fatty acids [43] | Stratum corneum | Tape stripping | Liquid chromatography mass spectrometry | Unclear |
| Unsaturated free fatty acids [43] | Stratum corneum | Tape stripping | Liquid chromatography mass spectrometry | No |
| Ceramide (AdS) [43] | Stratum corneum | Tape stripping | Liquid chromatography mass spectrometry | No |
| Ceramide (EOdS) [43] | Stratum corneum | Tape stripping | Liquid chromatography mass spectrometry | Unclear |
| Ceramide (EOP) [43] | Stratum corneum | Tape stripping | Liquid chromatography mass spectrometry | Unclear |
| Hydroceramide [28] | Stratum corneum | Stripping with cyanoacrylate resin | Thin layer chromatography | Yes |
| Wax [28] | Stratum corneum | Stripping with cyanoacrylate resin | Thin layer chromatography | Yes |
| Desmoglein 1[32] | Stratum corneum | Varnish stripping | SDS-page, western blotting | Yes |
| Plakoglobin [32] | Stratum corneum | Varnish stripping | SDS-page, western blotting | Yes |
| N(6)-carboxymethyl-lysine activity [42] | Stratum corneum | Cyanoacrylate skin surface stripping | Enzyme-linked immunosorbent assays | Yes |
| Interleukin-1α activity [42] | Stratum corneum | Cyanoacrylate skin surface stripping | Enzyme-linked immunosorbent assays | Unclear |
| Superoxide dismutase activity [42] | Stratum corneum | Cyanoacrylate skin surface stripping | Enzyme-linked immunosorbent assays | Unclear |
| Caseinolytic activities [38] | Stratum corneum | Tape stripping | Protease assay | Yes |
| Chymotrypsin-like activities [38] | Stratum corneum | Tape stripping | Protease assay | Yes |
| Trypsin-like activities [38] | Stratum corneum | Tape stripping | Protease assay | Yes |
| Histamine [41] | Compounds dissolved from stratum corneum | Collecting swabs | Liquid chromatography mass spectrometry | Yes |
| Glutathione [41] | Compounds dissolved from stratum corneum | Collecting swabs | Liquid chromatography mass spectrometry | Unclear |
| Melondialdehyde [41] | Compounds dissolved from stratum corneum | Collecting swabs | Liquid chromatography mass spectrometry | Yes |
| Natural moisturising factors [41] | Compounds dissolved from stratum corneum | Collecting swabs | Liquid chromatography mass spectrometry | Yes |
| Citrulline [33] | Stratum corneum | Scraping off the skin with a glass slide | High performance liquid chromatography | Yes |
| Gamma-aminobutyric acid [37] | Stratum corneum | Tape stripping | High performance liquid chromatography | Unclear |
| Carboxylic acid [38] | Compounds dissolved from stratum corneum | Collecting swabs | Fourier transform infrared spectroscopy | Yes |
| IL-1ra/IL-1β [36] | Compounds dissolved from stratum corneum | Collecting swabs | Enzyme-linked immunosorbent assays | Yes |
| Interleukin-8 [36] | Compounds dissolved from stratum corneum | Collecting swabs | Enzyme-linked immunosorbent assays | Yes |
| (Pro)filaggrin [37] | Stratum corneum | Tape strippings | Western blotting and densitometricanalyses | No |
| Bleomycin hydrolase [37] | Stratum corneum | Tape strippings | Western blotting and densitometricanalyses | Yes |
| Total proteins [41] | Compounds dissolved from stratum corneum | Collecting swabs | Liquid chromatography mass spectrometry | Yes |
| Annexin A2 [34] | Stratum corneum | Varnish stripping | Electrophoresis, western blot, liquid chromatography mass spectrometry | Yes |
| Phosphatidylethanolamine-binding protein 1 [34] | Stratum corneum | Varnish stripping | Electrophoresis, western blot, liquid chromatography mass spectrometry | Yes |
