# Supplementary material for: Molecular characterization of xerosis cutis: A systematic review
Source: PLoS One. 2021 Dec 16;16(12):e0261253. doi: 10.1371/journal.pone.0261253 (PMC8675746; doi:10.1371/journal.pone.0261253)
Supplement: S1 File — (PDF) [file pone.0261253.s005.pdf]

To enable PROSPERO to focus on COVID-19 registrations during the 2020 pandemic, this registration record was automatically published exactly as submitted. The PROSPERO team has not checked eligibility.

Citation

Ruhul Amin, Anna Lechner, Annika Vogt, Jan Kottner. Molecular characterization of xerosis cutis: A systematic review. PROSPERO 2020 CRD42020214173 Available from:
https://www.crd.york.ac.uk/prospero/display_record.php?ID=CRD42020214173

Review question

What is the molecular characterization of xerosis cutis (dry skin)? How is the expression of molecular markers associated with clinical sings of dry skin? Are there differences between different dry skin etiologies?

Searches

Resources:
-'MEDLINE','EMBASE' and 'Biological abstracts' databases will be searched concurrently via OvidSP.

- Reference lists of all potentially eligible publications will be screened.

-'Cited Reference Search' search in 'Web of Science' will be conducted using the studies that meet the eligibility criteria.

-The search will be updated once near to the end of the review.

Search strategy

https://www.crd.york.ac.uk/PROSPEROFILES/214173_STRATEGY_20201013.pdf

Types of study to be included

Inclusion criteria
- Primary studies reporting quantitative data,

- Humans/ in vivo,

- Subjects, patients with symptoms of dry skin/ xerosis

- Xerosis caused by internal/systemic changes/ diseases (e.g. metabolic disorders, hormonal changes, nutritional deficits, drug-related chronological ageing)

- Clear definition and/or reporting of symptoms and/or severity of dry skin (e.g. using a classification)

- Clear reporting of skin areas

- Clear quantitative reporting of biological markers and analytical methods.

- Clear reporting of age,

- All languages,

- All age groups,

- Publication date: from January 1990 until October 2020

Exclusion criteria

- Reviews, letters, editorials, personal opinions, posters and conference abstracts

- Pre-clinical/animal studies, in vitro studies

- Skin dryness due to external causes, such as exposures to irritants, allergens, pathogens, topical treatments.

- Dermatological diseases such as inflammatory skin diseases (dermatitis, psoriasis, eczema and/ or comparable conditions).

Condition or domain being studied
Xerosis cutis (skin dryness) is a highly prevalent dermatological condition caused by impairments of the skin barrier function and a lack of moisture retention capacity. Deregulated molecular pathways maybe associated with dry skin. Understanding their functional association with the orchestration of the symptoms is important for a better understanding of the pathological processes of skin dryness in different diseases and conditions.

Participants/population
Subjects with symptoms of dryskin, from birth to end of life.

Intervention(s), exposure(s)
Molecular markers of dry skin associated with a systemic/internal disease or condition.

Comparator(s)/control
Molecular markers of dry skin associated with another systemic/internal disease or condition.

Main outcome(s)
Quality and quantity of molecular markers associated with dry skin.

Measures of effect

Timing: cross-sectional and longitudinal studies.
Effect measures: quantification of molecular markers in units according to the described procedure.

Additional outcome(s)
Not Applicable

Measures of effect

Not Applicable

Data extraction (selection and coding)
The retrieved titles and abstracts following the inclusion criteria will be independently screened by the reviewers (RA and AL). Any difference in opinions between reviewers will be resolved by consensus or by a third reviewer (Jan Kottner, Annika Vogt). Full text articles of all potentially eligible studies will be independently checked for eligibility by the reviewers (RA and AL). Again, disagreements will be adjudicated through consensus or by discussion with a third author (Jan Kottner, Annika Vogt). Data extraction from included studies will be accomplished by the reviewers (RA and AL) by using a standardized data extraction form containing 1.Author; 2. Year; 3. Study design; 4. Number of participants; 5. Age; 6. Gender; 7. Country/ Ethnicity; 8. Severity of skin dryness; 9. Signs of dry skin and scoring method; 10. Sample; 11. Sampling Technique; 12. Method of analysis; 13. Skin areas; 14. Molecular Marker measured; 15. Results; 16. Quantification units; 17.Comments.

Risk of bias (quality) assessment
No formal risk of bias assessment will be conducted.

Strategy for data synthesis
Study results will be summarized descriptively. Associations between molecular markers and dry skin will be described within and between groups of different causes of dry skin.

Analysis of subgroups or subsets

Subgroup analysis will be conducted for different causes of dry skin.

Contact details for further information

Ruhul Amin
ruhul.amin@charite.de

Organisational affiliation of the review

Charité-Universitätsmedizin Berlin
www.charite.de
https://crc.charite.de

Review team members and their organisational affiliations

Mr Ruhul Amin. Charité-Universitätsmedizin Berlin
Ms Anna Lechner. Charité-Universitätsmedizin Berlin
Professor Annika Vogt. Charité-Universitätsmedizin Berlin
Professor Jan Kottner. Charité-Universitätsmedizin Berlin

Type and method of review

Systematic review

Anticipated or actual start date

29 September 2020

Anticipated completion date

01 September 2021

Funding sources/sponsors

Clinical Research center for Hair and Skin Science, Department of Dermatology and Allergology, Charité-Universitätsmedizin Berlin.

Conflicts of interest

Language

English

Country

Germany

Stage of review

Review Ongoing

Subject index terms status

Subject indexing assigned by CRD

Subject index terms

MeSH headings have not been applied to this record

Date of registration in PROSPERO

13 November 2020

Date of first submission

13 October 2020

Stage of review at time of this submission

Stage	Started	Completed
Preliminary searches	Yes	Yes
Piloting of the study selection process	Yes	No
Formal screening of search results against eligibility criteria	Yes	No
Data extraction	No	No
Risk of bias (quality) assessment	No	No
Data analysis	No	No

The record owner confirms that the information they have supplied for this submission is accurate and complete and they understand that deliberate provision of inaccurate information or omission of data may be construed as scientific misconduct.

The record owner confirms that they will update the status of the review when it is completed and will add publication details in due course.

Versions

13 November 2020
